# Hydrogel: A Potential Material for Bone Tissue Engineering Repairing the Segmental Mandibular Defect

**DOI:** 10.3390/polym14194186

**Published:** 2022-10-05

**Authors:** D S Abdullah Al Maruf, Yohaann Ali Ghosh, Hai Xin, Kai Cheng, Payal Mukherjee, Jeremy Micah Crook, Gordon George Wallace, Travis Jacob Klein, Jonathan Robert Clark

**Affiliations:** 1 Integrated Prosthetics and Reconstruction, Department of Head and Neck Surgery, Chris O’Brien Lifehouse, Camperdown 2050, Australia; 2 Central Clinical School, Faculty of Medicine and Health, The University of Sydney, Camperdown 2050, Australia; 3 Royal Prince Alfred Institute of Academic Surgery, Sydney Local, Camperdown 2050, Australia; 4 Biomedical Innovation, Chris O’Brien Lifehouse, Camperdown 2050, Australia; 5 School of Medical Sciences, Faculty of Medicine and Health, The University of Sydney, Camperdown 2050, Australia; 6 Sarcoma and Surgical Research Centre, Chris O’Brien Lifehouse, Camperdown 2050, Australia; 7 ARC Centre of Excellence for Electromaterials Science, The University of Wollongong, Wollongong 2522, Australia; 8 Intelligent Polymer Research Institute, AIIM Facility, The University of Wollongong, Wollongong 2522, Australia; 9 Illawarra Health and Medical Research Institute, The University of Wollongong, Wollongong 2522, Australia; 10 Centre for Biomedical Technologies, Queensland University of Technology, Kelvin Grove 4059, Australia

**Keywords:** bone tissue engineering, hydrogel, mandibular defect, 3D printing, scaffolds

## Abstract

Free flap surgery is currently the only successful method used by surgeons to reconstruct critical-sized defects of the jaw, and is commonly used in patients who have had bony lesions excised due to oral cancer, trauma, infection or necrosis. However, donor site morbidity remains a significant flaw of this strategy. Various biomaterials have been under investigation in search of a suitable alternative for segmental mandibular defect reconstruction. Hydrogels are group of biomaterials that have shown their potential in various tissue engineering applications, including bone regeneration, both through in vitro and in vivo pre-clinical animal trials. This review discusses different types of hydrogels, their fabrication techniques, 3D printing, their potential for bone regeneration, outcomes, and the limitations of various hydrogels in preclinical models for bone tissue engineering. This review also proposes a modified technique utilizing the potential of hydrogels combined with scaffolds and cells for efficient reconstruction of mandibular segmental defects.

## 1. Introduction

Segmental defect of the mandibles can be instigated by resection of malignant carcinoma, trauma, osteomyelitis, osteonecrosis of jaws [1]. Osseous free flaps have revolutionised mandibular reconstruction with success rates exceeding 95%; however, donor site morbidity is a significant problem for patients who undergo these long and complex surgeries. Bone tissue engineering (BTE) employs novel strategies for reconstructing critical sized bone defects to improve the quality of life for patients suffering bone loss [2]. In the context of segmental mandibular defects, BTE aims to avoid or minimise donor site morbidity via an innovative regenerative platform [3].

BTE structures need to be mechanically stable and integrate with neighboring bone to achieve a durable reconstruction. The immediate and long-term goals typically differ, with osteogenic strategies employed to support bone growth that ultimately achieves the desired mechanical stability and in the case of injury, regenerative capacity. Various biomaterials including polycaprolactone (PCL), polyether ether ketone (PEEK), polyetherketone ketone (PEKK), ceramics, metals, polymethylmethacrylate (PMMA), polyglycolic acid (PGA), and polylactic acid (PLA) hydrogels have been extensively researched for bone regeneration [4]. An ideal biomaterial must be safe, biocompatible, cytocompatible (non-cytotoxic or do not cause harm to the cells), bioinert (when implanted, has minimal interaction with its surrounding tissue), bioactive (has a biological effect), biostable, and biodegradable [5]. However, it is unlikely that a single biomaterial will fulfil all these requirements.Hence, hybrid scaffolds utilizing the favorable characteristics of different materials are being investigated, including natural and synthetic polymers [6,7].

Recent research has focused on the critical role of the extracellular matrix (ECM) in bone repair. Hydrogels are a type of polymeric network that is soft and highly hydrated [8,9]. They provide a three-dimensional (3D) environment resembling tissue ECM and an appropriate milieu for cellular adhesion, proliferation, migration, and differentiation [10]. When combined with the appropriate structural support, the gels may be able to augment repair of critical-sized bone defects.

The features of a given biomaterial are determined by its physical, chemical, structural (nano-, micro-, macro-), and mechanical properties, as well as biocompatibility, biodegradability, and related hydrophilicity [11,12]. There are two distinct complimentary enablers of BTE scaffolds: osteoinduction and osteoconduction. Osteoinduction requires the scaffold to provide structural support for progenitor cells and a biological environment that favours osteogenic differentiation, proliferation, and mineralization [11]. Significant progress in the field of biomaterials has been evident over recent decades [4]. However, segmental mandibular defects of the mandible are a particularly challenging clinical scenario because of the microorganism-rich environment, minimal soft tissue cover, the consistent requirement for adjuvant radiotherapy, complex geometry, specialised anatomical structures (teeth), and high-stress axial and non-axial (cantilever) loading [4]. For critical sized bone defects, the medium to long-term mechanical characteristics of scaffolds is particularly important, as it may take months for bone to grow across oral cancer-related mandibular defects that are commonly 6–10 cm in length without osteoinduction.

This review article provides an overview of the fundamental properties of various types of hydrogels with a focus on the design, fabrication, challenges, and potential application strategies pertaining to the reconstruction of segmental mandibular bone defects.

## 2. Bone Composition

Bone is a composite connective tissue that undergoes continuous remodelling in response to biochemical and physical factors [13]. It is comprised of an organic and inorganic matrix, cells, and water [14]. The primary functions of bone are to provide structural support, serve as a site of attachment for muscles, mineral storage, and haematopoiesis [15].

The biomechanical properties of bone vary due to two regions of distinct structure. Cortical bone, the outer layer, is characterised by dense rigid lamellar bone mostly arranged according to the direction of mechanical stress [13,16]. Cortical bone is lined by periosteum on the outside surface and endosteum on the inside surface. Trabecular bone, encased within cortical bone, has a highly porous framework, and contains many interconnecting spaces (marrow space) lined with an endosteum [13]. The greater porosity of trabecular bone makes it less resistant to sudden, high impact forces compared to cortical bone [16]. Therefore, not all bones have the same proportion of cortical to trabecular bone mass. For example, the mandible has a cortical: trabecular ratio of 80:20, compared to 25:75 for vertebra, to accommodate the intense cantilever forces required for mastication [13,17].

Bone ECM is a highly organised structure that supports the mechanical and physiological demands of bone. There is a preponderance of Type 1 Collagen in the organic component, supplemented by non-collagenous proteins and growth factors such as Bone Morphogenic Proteins (BMP) [18]. The inorganic component is predominantly calcium phosphate crystallised as hydroxyapatite, in addition to various bicarbonate, sodium, and potassium salts [19]. This inorganic component also acts as a mineral reservoir under endocrine control [13].

Collagen fibrils are organised in one of two arrangements: woven or lamellar [20]. Woven tissue is an immature arrangement of bone characterised by disorganised collagen and a large volume of bone cells. This forms rapidly following injury before slowly transforming into lamellar bone [13]. Lamellar tissue is a mature arrangement of bone characterised by highly organised collagen deposited in alternating orientations in parallel concentric lamellae, providing stronger biomechanical properties than woven bone [13].

**Figure 1:**
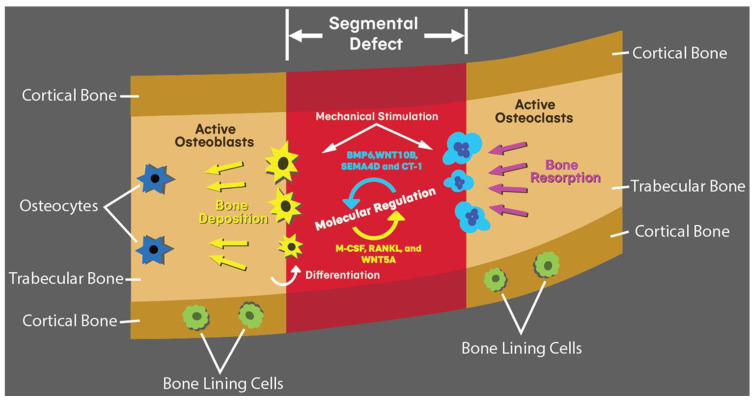
Actions of osteoclasts, osteoblasts and bone lining cells in the context of a segmental mandibular defect. Selected molecular mechanisms of regulation between osteoblasts and osteoclasts include BMP6, WNT10B, SEMA4D, CT-1, M-CSF, RANKL, and WNT5A.

There are four distinct cell types that contribute to bone growth as depicted in Figure 1: osteocytes, osteoblasts, osteoclasts, and bone-lining cells [14]. Osteocytes are the most common cell (90–95%) in mature bone and have cytoplasmic processes that mediate intercellular signalling [21]. Osteoblasts are derived from mesenchymal stem cells (MSCs). Their primary function is to secrete osteoid into the bone matrix and further differentiate into osteocytes [22]. Bone-lining cells are a latent form of osteoblast that prevent resorption of bone surfaces [23]. Osteoclasts are derived from haemopoietic stem cell lineage and facilitate mineral resorption during remodelling [24].

Cellular activity of these cells is regulated by juxtacrine and paracrine signalling. Osteoclast activation is achieved by osteoblast expression of macrophage -colony stimulating factor M-CSF, receptor activator of nuclear factor kappa-β ligand (RANKL), and Wnt gene family 5A (WNT5A), while the reverse is activated by osteoclast expression of bone morphogenetic protein 6 (BMP6), Wnt gene family 10B (WNT10B), Semaphorin 4D (SEMA4D) and Cardiotrophin-1 (CT-1) [25].

## 3. Types of Segmental Mandibular Defects and Principles of Fracture Healing and Osteogenesis

**Figure 2:**
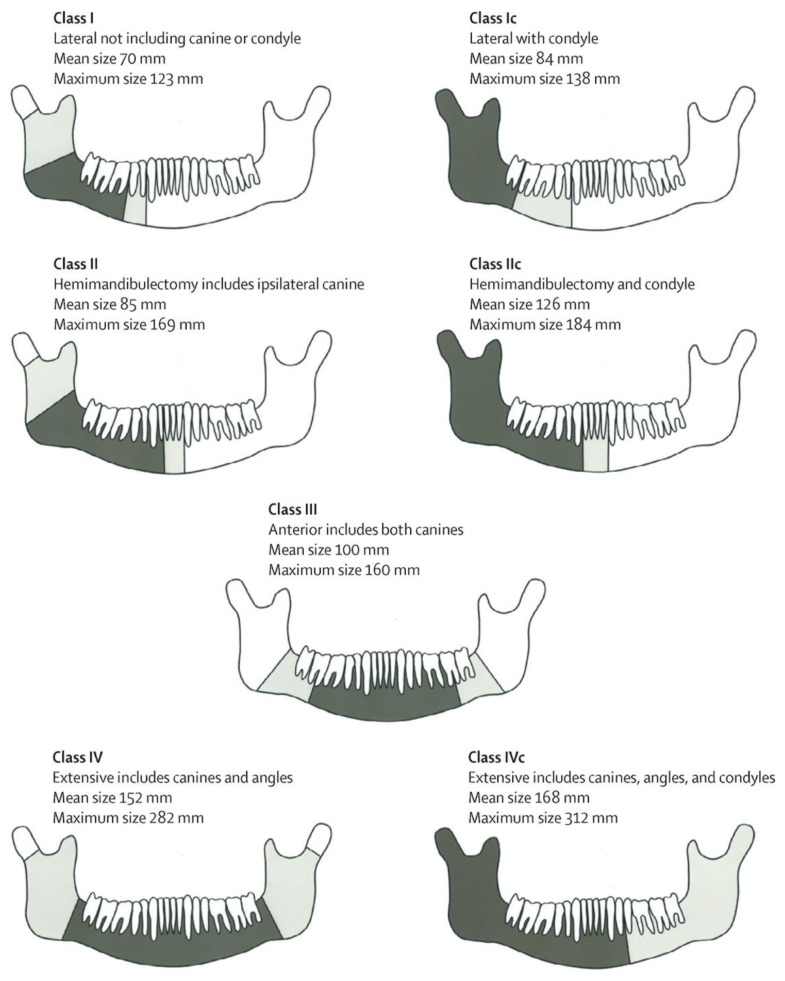
Proposed classification of mandibular defects. Mean defect size (dark shading); total extent of mandibular defect (light shading). Reprinted with permission from Elsevier [30].

The mandible is a complex bone with unique characteristics that distinguish it from long bones and the axial skeleton. Segmental mandibulectomy is the term used to describe the surgical resection of a portion of the mandible that leads to discontinuity. The specialised composition and structure of the mandible with non-axial loading makes it a unique challenge for BTE. Although several classification systems have been developed, none are universally accepted [26,27,28,29]. Brown et al. (2016) is a widely cited tool for classifying mandibular defects and more specifically to distinguish between different extents of resection (Figure 2) [1,28]. Brown’s classification is based on the four corners of the mandible and two specialised structures: the condyle and dentition [30]. The classification consists of four defect classes: Class I (limited to the angle, excludes canines or condyles), Class II (includes the angle and ipsilateral canine), Class III (limited to both canines, excludes the angle) and Class IV (includes both canines and at least one angle). Notably, variations exist to Classes I, II, and IV, respectively referred to as Class Ic, IIc, and IVc, that include the resection of the ipsilateral condyle [31].

Bone is a dynamic tissue that undergoes continual change in response to use, damage, and other external and internal stimuli [14]. The principles of bone healing and growth have traditionally been described by Wolff’s Law, such that the orientation of long bone healing is determined by mechanical stress [32,33]. However, *Mechanostat Theory* explains the life-long changes in structure and mass with relation to mechanical use and the varying local deformation of bone.

*Mechanostat Theory* involves three mechanisms: growth, modelling and re-modelling [34]. In adults, the major form of osteogenesis for irregular bones like the mandible is intramembranous ossification [35]. During intramembranous ossification, MSCs differentiate into osteoblasts that secrete an unmineralised, collagen-proteoglycan rich osteoid that binds calcium ions. During this process, blood vessels are encased within the tissue [19]. Gradual adjustments to the overall shape of bone that occur in response to mechanical stimuli are termed modelling [13]. However, bone remodelling is the process of coordinated matrix resorption and deposition in response to microdamage of the tissue [13]. The coordinated function of both osteoblasts and osteoclasts in remodelling is guided by mechanotransduction–the conversion of mechanical stimulus into biochemical stimulus [19]. Notably, the rate of bone turnover is greater in the mandible than in the appendicular skeleton [36]. Two explanations have been proposed: (i) the mandible embryologically arises from the neuroectoderm and not the mesoderm, and (ii) the major role of intramembranous ossification instead of endochondral ossification for osteogenesis [37].

Maxillofacial fractures differ to other fractures of the skeleton due to the presence of dentition and associated anatomical structures. In healthy adults, the mandible is weakest at the condyles, and progressively stronger towards the midline [38]. Fracture healing can occur primarily or secondarily. Primary healing occurs infrequently, only for small defects, and involves the direct ossification of the fracture by osteoblasts in the absence of callus formation [39]. However, secondary fracture healing involves both intramembranous and endochondral ossification [40]. The initial inflammatory stage is characterised by haematoma formation and the local secretion of proinflammatory molecules [19]. In the repair stage, MSCs are recruited from surrounding tissue to differentiate into chondrocytes and secrete a temporaneous cartilaginous callous that undergoes endochondral ossification [19]. The final stage of fracture healing is remodelling with the cartilaginous callous being resorbed, a mineralised matrix deposited, and normal bone turnover continued [40]. Because fracture healing is completed by remodelling, healing of the post-extraction or partially edentulous mandible occurs in the absence of normal mastication forces and will result in relative bone resorption [41].

## 4. Current Techniques for Reconstructing Segmental Mandibular Defects

The goal of oromandibular reconstruction is to restore both form and function [41]. Three surgical strategies are currently used for reconstruction of segmental mandibular defects: vascularised bone grafts, non-vascularised bone grafts and distraction osteogenesis.

**Figure 3:**
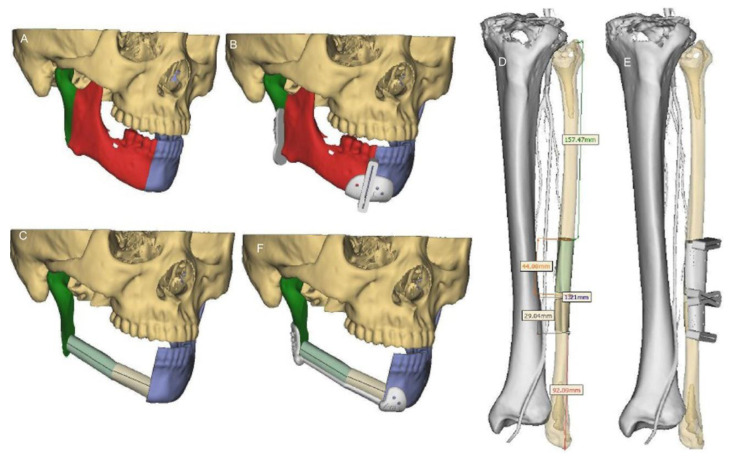
(**A**,**B**) Three-dimensional mandibular resection and design of the mandibular resection guide for a patient with intra-osseous carcinoma of the mandible, using the virtual surgical planning software. (**C**) Virtual planning of the fibular inset. (**D**,**E**) Automatic virtual planning of fibula flap length, osteotomy angle, and osteotomy guide. (**F**) Virtual planning of the mandibular reconstruction template Reprinted with permission from Elsevier [44].

Vascularised bone grafts, also known as osseous free flaps, can include skin (osteocutaneous) or muscle (osteomyogeneous). Osteocutaneous free flaps are the current gold-standard for reconstructing segmental mandibular defects, with the fibula bone being the most common donor site (Figure 3) [1]. Osteocutaneous free flaps involve the harvest of tissue from an autologous donor site on a vascular pedicle and transplantation of the tissue using microsurgery to anastomose the artery and vein of the flap to suitable vasculature at the recipient site. Osseointegrated implants can be placed in the bone to facilitate dental rehabilitation via a dental prosthesis secured to the implants. In recent years, virtual surgical planning has optimised the placement of bone to enhance functional and cosmetic outcomes [41].

**Figure 4:**
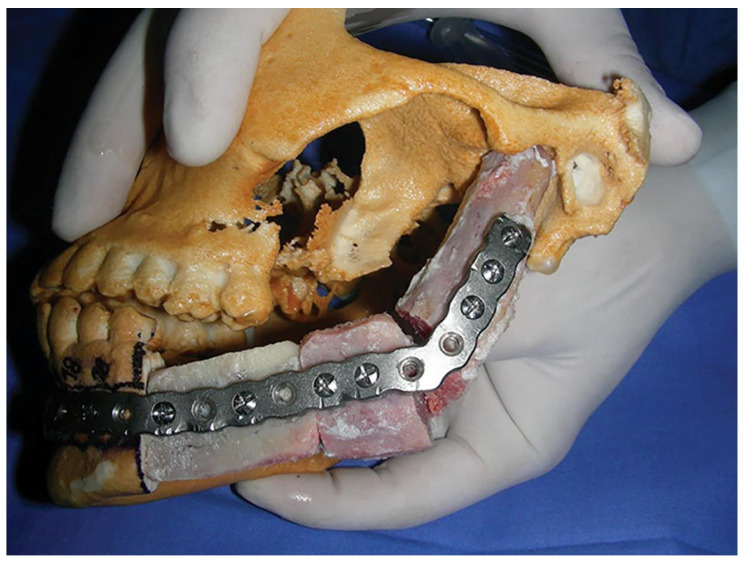
Non-vascularised iliac bone graft adapted to premolded reconstructing plate. Reprinted with permission from [45].

Non-vascularised bone grafts include autografts, allografts, or xenografts that are used to fill the defect (Figure 4) [42]. However, non-vascularised bone grafts are less versatile and less reliable than vascularised bone flaps. Bone grafts need complete soft tissue coverage to separate the graft from the oral cavity for success; this is often impossible in the setting of oral cancer. Furthermore, both radiotherapy and longer graft lengths are associated with high failure rates [43].

Distraction osteogenesis is a procedure that induces rapid bone growth by creating a fracture in the mandible and gradually increasing its width. Distraction osteogenesis is commonly used in orthognathic surgery for small length discrepancies. However, in the setting of oral cancer, this technique is limited for similar reasons to non-vascularised bone grafts [41].

## 5. Types of Hydrogels

Hydrogel-based scaffolds can be derived from multiple sources, including natural and synthetic polymers. Polymers of natural origin are categorized as *protein-based* (e.g., gelatin, collagen, fibrin, and silk fibroin) or *polysaccharide-based* (e.g., hyaluronic acid, chondroitin sulphate (CS), alginate, and chitosan) [46]. As reported, protein-based hydrogels have many advantages including biocompatibility, tune-able mechanical properties, biodegradability, and comparable chemical and mechanical structures to ECM. They can be crosslinked to form gel networks via protein chains unfolding, hydrogen bonds, covalent crosslinking, etc. Additionally, proteolytic enzymes are able to degrade the hydrogel network, making protein-based hydrogels a promising candidate for tissue engineering and drug delivery [47]. Similar to protein-based hydrogels, those prepared from polysaccharides also exhibit biocompatibility, hydrophilicity, excellent physiochemical and biological performance, and the ability to promote cell growth and differentiation. However, some significant shortcomings also limit the realisation of polysaccharide-based hydrogels in clinical practice, including low purity and limited prospects for in vivo crosslinking strategies [48].

Hydrogels can also be categorised by their crosslinking mechanism such as covalent, physical or hybrid crosslinking. Physical crosslinks include hydrogen bonds, ionic chelation, host-guest interaction, electrostatic complex, and hydrophobic interaction [49]. Hydrogels prepared by covalent crosslinking exhibit mechanical stability but structural and damage irrecoverability. On the other hand, hydrogels synthesised with physical crosslinks demonstrate mechanical recoverability, since the physical bonds are reversible and able to reconstruct following mechanical fracture. Significant characteristics of physically crosslinked hydrogels are stress relaxation and fatigue which may limit their applications in load-bearing medical devices [49,50].

### 5.1. Protein-Based Hydrogels

Collagen is a principal component of the bone ECM and an attractive option for biomedical applications because it is robust, abundant, biocompatible and its physiochemical properties are maintained in vitro. Furthermore, it undergoes aggregation and self-assembly through the process of non-enzymatic glycation and crosslinking [51,52]. Collagen dissolves in acid and can be transformed into a hydrogel with a self-assembled triple helix structure at 37 °C in a neutral environment [52]. Although collagen gels tend to be very soft, with a compressive modulus in the order of 1 kPa, this stiffness can be tailored by changing temperature, pH, concentration, or adding further crosslinking steps, and can be used as a vehicle to deliver cells and growth factors in the desired recipient site [51,53]. As a native component of ECM, type 1 collagen has proven to be a better choice than non-native components such as hyaluronic acid for the function and proliferation of osteoblasts. A 3D-printable construct consisting of osteoblast-encapsulated collagen and chondrocyte-encapsulated hyaluronic acid has been developed with good cell viability which were retained for 14 days [54] One of the obvious drawbacks for collagen is slow gelation time. As reported, type 1 collagen takes around 30 min to form a gel at 37 °C. This may lead to inhomogeneous cell distribution after the bioprinting process. This is why collagen is usually printed with support materials and embraced into other hydrogel systems to improve its gelling behaviours, mechanical stability, and cell homogeneity [55]. With the latest development of extrusion printing in suspension baths, collagen hydrogels of relatively low viscosity can be printed in a bath filled with high thixotropy fluid, which enables the printing of collagen hydrogels with high viscosity and negates the need for fast gelation. Meanwhile, several in vitro approaches have been developed to mineralise collagen to facilitate the formation of hard tissues [56].

Gelatin is a protein mixture produced from the hydrolysis of collagen. It has several functional groups that can be utilised directly or further modified for crosslinking, making it a popular candidate for applications such as tissue engineering matrices and drug delivery systems [56,57]. Gelatin is highly water soluble at room temperature, does not induce an immune response, and exhibits amphoteric behaviour [58,59]. The gelling mechanism of gelatin in water is well-studied and found to be associated with temperature-induced conformational change of the gelatin molecules from a random coil state at higher temperature to the helix state leading to collagen fold when it is cooling [60,61]. Gelatin is known to be mechanically instable and possesses significant enzyme digestibility, which may prohibit the applications of gelatin. These drawbacks may be overcome by the chemical crosslinking of gelatin with a carbodiimide reaction. The resultant gelatin scaffolds demonstrate promising osteoinductive effect on human mesenchymal stem cells with both inorganic hydroxyapatite and organic BMP-2 peptide signalling [62]. One of the most documented gelatin derivatives is gelatin methacryloyl (GelMA) which can be synthesized by a reaction between gelatin and methacrylic anhydride. The methacryloyl substitute enables the GelMA network to be photo-crosslinkable. By adjusting the degree of methacryloyl substitutes, the density of photo-crosslinking can be altered to tailor the modulus or stiffness of the hydrogel scaffold [63], which exerts profound influences on the differentiation of mesenchymal stem cells [64]. Due to its good cell adhesion, biocompatibility, and adjustable mechanical properties, GelMA has been found to be useful in many tissue engineering applications [65].

Fibrin is another protein-based hydrogel with potential as a cell delivery vehicle and injectable scaffold [66]. Fibrin is a normal part of the tissue repair response and can be generated from a patient’s blood to obtain the natural precursors of fibrin hydrogels including fibrinogen and thrombin. In a knee injury model, fibrin has been utilized with hyaluronic acid-based gels to transport chondrocytes [67]. One significant advantage of fibrin is that it is a natural scaffold material which plays crucial roles in providing temporary structural support and facilitate haemostasis following tissue damage and injury [68]. In one research work, fibrin has been utilised to fabricate an Adipose Derived Stem Cell (ADSC) containing scaffold to regenerate mandibular bone defecta in a rabbit model, and significant increases in the thickness of new cortical bone growth was discovered for fibrin glue scaffolds which incorporate ADSCs [69].

Another sub-group of protein-based hydrogel is silk fibroin which is extracted from silkworm silk. Silk fibroin possesses excellent mechanical properties, biocompatibility, and bio-absorbability. Since it can be dissolved in aqueous solution, it can be easily processed into various forms for different applications in tissue engineering [70]. Due to the high mechanical toughness, silk fibroin scaffolds are suitable for bone implants. A porous silk fibroin scaffold has been used to fabricate biomaterials with human mesenchymal stem cells. Results have demonstrated the capability of this scaffold system to initiate de novo bone growth [71].

### 5.2. Polysaccharide-Based Hydrogels

Hyaluronic acid is a glycosaminoglycan prevalent in native ECM and specialised body fluids, such as synovial fluid and ocular vitreous humor [72]. Hyaluronic acid possesses high viscoelasticity and space filling properties [52]. As such, it has been used extensively in the cosmetic industry as an injectable filler for the skin [46]. and in wound healing applications [73,74].

Alginate is derived from brown seaweed and has been used in clinical practice as a wound dressing [43,75,76,77]. Alginate molecules contain M blocks (1,4 linked β-D-mannuronic acid) and G blocks (α-L-guluronic acid). Since the G blocks can form ionic chelation with divalent cations such as Ca^2+^, alginates can be ionically crosslinked to form a gel network that is biocompatible, biodegradable, and easily modified [78,79]. The crosslinking junction of alginate hydrogels between the G block and cations, such as Ca^2+^, takes the form of an “egg-box” which can be broken and reconstructed, thereby showing damage reversibility [80]. One example is so-called ionic-covalent alginate/polyacrylamide hybrid hydrogels. In this hydrogel, alginate is physically crosslinked by Ca^2+^ while polyacrylamide is covalently crosslinked. The resultant hydrogel system exhibits high mechanical toughness (thousands of J/m^2^) and damage recoverability. Therefore, this hydrogel is a promising candidate for load-bearing applications such as BTE scaffolds and human cartilage substitutes [78]. 3D printable alginate/polyacrylamide interpenetrating hydrogels have been developed with UV irradiation to polymerize the precursor gel ink. The obtained gels were further reinforced by being immersed in CaCl_2_ solutions. This hydrogel system exhibited highly enhanced mechanical properties and holds great potential for applications in bone tissue engineering and soft robotics [81].

### 5.3. Synthetic Hydrogels

Synthetic polymers (SYPs) have numerous benefits over their natural-origin equivalents. These include a wider range of mechanical characteristics [82], a process-controllable batch-to-batch consistency, as well as a defined chemistry [83]. These properties allow for mass production while maintaining quality, which is critical in biological applications [83]. Poly(2-hydroxyethyl methacrylate) (PHEMA), poly(ethylene glycol) (PEG), poly(vinyl alcohol) (PVA), poly(N-isopropylacrylamide) (PNIPAM) [84], and polyacrylamide (PAM) are examples of synthetic polymers currently utilised in hydrogels [85]. Synthetic polymers, unlike natural materials, have basic structural units allowing polymer features such as porosity, degradation time, and mechanical capabilities to be tailored to specific applications [6]. Synthetic polymers offer reliable material sources and lengthy shelf lives, allowing them to be mass-produced with low risk of immunogenicity [86]. One of the first synthetic biomedical hydrogels was PHEMA [87], created from radical chain polymerization of 2-hydroxyethyl methacrylate [52]. Hydrophilic polymers, such as PHEMA and PEG, resist protein adsorption and cell adhesion. They can be modified to include cell adhesion sites and enzyme cleavage sites to improve their interactions with cells [88]. These hydrogels are frequently employed as structural scaffolds for guided tissue growth or cellular encapsulation as cell delivery vehicles [52,89,90,91,92,93]. Other synthetic hydrogels, such as PVA, possess highly elastic mechanical properties that can influence cellular orientation or matrix synthesis [94]. As a thermal-responsive polymer that undergoes gelation at around physiological temperature, PNIPAM hydrogel scaffolds have also received considerable attention. Their mechanical and physiochemical properties can be modified by optimising the synthesis parameters and crosslinking approaches to fit the applications of cartilage regeneration and tissue engineered scaffolds [95].

## 6. Properties of Hydrogels for BTE

Hydrogels have a very high-water content (90–99% *w*/*w*) and are characteristically viscoelastic. This mechanical property is related to their water content as well as the crosslinking properties of the backbone polymer (the number, type, and size of the cross-linked molecules), which also determines their porosity, swelling, and degradation [96,97,98,99,100,101]. Polymeric concentration also affects the ability of the scaffolds to integrate proteins and prevent cell aggregation [102].

The isometric tension of ECM varies according to tissue type [64] and the cellular response to these tensions and stresses can also be different, leading to structural modifications mediated through gene expression [103]. As a result, tissue-specific mechanical properties may be required when designing hydrogel scaffolds. Hydrogel stiffness influences cellular response, for example differentiation of MSCs [104]. In biological conditions, hydrogels can swell and hold large volumes of water. The molecular weight of a polymer and the type of crosslinking are the essential elements that determine the swelling behaviour of hydrogels [105]. Drug release in typical hydrogel systems is primarily accomplished by hydrogel swelling/contraction and drug diffusion via the polymer network [106].

## 7. Design Strategies for Hydrogel-Based Scaffolds Used in BTE

### 7.1. Mechanical Properties

Despite favourable biocompatibility, naturally derived hydrogels possess some limitations such as low mechanical strength, uncontrollable biodegradation rate, and potential immunogenic response [107]. These limitations hinder their clinical translation. Synthetic polymers offer tuneable microstructure, extended robustness, and improved mechanical strength, however, they have limited biological activity [108]. This has led to the development of hybrid hydrogel-based scaffolds which utilise the favourable features from both natural and synthetic sources. As discussed earlier, the poor mechanical properties of hydrogels can be improved by covalent crosslinking and incorporating secondary materials. Although covalent crosslinking improves the mechanical properties, this irreversible process may also be cytotoxic [109]. Several other hydrogel synthesis strategies have also been developed to alter the mechanical properties such as double network hydrogel (DN) [110], clay-polymer nano-composite hydrogels [111], slide-ring hydrogels [112], hydrogen-bond toughened hydrogels [113], and ionic-covalent crosslinking hybrid hydrogels [78].

Two polymers, natural and/or synthetic, may be interpenetrated to form a double network (DN) hydrogel where the two polymers are crosslinked independently but interpenetrated with each other, with the resultant hydrogel expressing desirable features from each polymer type [114]. Due to the special network topologies and energy dissipation mechanisms, these DN hydrogels have improved mechanical properties and provide more control over physical parameters than single polymer networks [115]. Co-polymeric hydrogels such as poly(ethylene glycol)-poly(e-caprolactone)-poly(ethylene glycol) (PECE) hydrogel are made up of monomers, each of which has at least one hydrophilic component that plays a vital role in the swelling behaviour [116].

Nano-composite hydrogels are polymeric networks with high water content that crosslink with one another and/or nanoparticles [117]. Nano-materials have been incorporated into the polymeric networks with a goal of enhancing the mechanical characteristics. Ceramic nano-particles (hydroxyapatite) [118,119], carbon-based (graphene) [120], and metallic nano-particles (gold and silver) [121,122] have been integrated into hydrogel networks to develop nanocomposites with desirable physical properties and functionality.

Slide-ring hydrogels contain polyethylene glycol chains threaded into hydroxypropyl-*α*-cyclodextrin (*α*-CD) rings. The rings are covalently connected to form the hydrogel network but slide along the macromolecular chains when the polymer network is subject to the external stress. In this way, the mechanical stress can be better distributed to the network to enhance its mechanical properties. This hydrogel system also exhibits fast damage recovery [112].

Both DN hydrogels, nano-composite hydrogels and slide-ring hydrogels exemplify the advancements that material scientists have made to improve the mechanical performance of hydrogels for load-bearing biomedical applications including tissue regeneration and replacement. As mentioned above, ionic-covalent alginate-polyacrylamide hybrid hydrogels prepared with recoverable Ca^2+^ ionic crosslinks exhibit several thousands of J/m^2^ of fracture energy and super stretchability (more than 10 times its original length). Indeed, this hydrogel system is thought to be a candidate for cartilage replacement [78]. By adjusting Ca^2+^ concentration and types of divalent cations, the stiffness of the hydrogel can also be tuned [79,123]. 3D printable tough hydrogel scaffolds have also been developed with adjustable mechanical properties, with these systems having great potential for bone tissue engineering and drug delivery applications [81,124].

### 7.2. Porosity

To facilitate cellular ingrowth, homogenous cell distribution, and matrix neovascularization, it is necessary for a scaffold to be highly porous and interconnectedwith a large surface area relative to volume [64,125]. Additionally, consideration of factors such as pore size, volume, size distribution, shape, and wall roughness are equally critical [12]. While internal diffusion is limited in hydrogels, porosity is an important physical element to enable nutrition and oxygen transport for cell viability [126]. In terms of osteoconduction, the optimal pore size is in the range of 200–350 µm [64,127,128,129,130,131]. Moreover, it has been recommended that scaffolds applied to BTE should be more than 90% porous [132]. Modifying the degree of crosslinking or adding porogen materials, such as non-crosslinked gelatin microspheres, can be used to control the porosity of the scaffolds [133].

Vascularization is an essential process in bone healing; it has been suggested that hydrogels should have a pore size between 50–300 µm to facilitate neovascularization [134,135]. The physical breakdown of hydrogels can facilitate cellular migration and vascular infiltration [136]. Ideally, the degradation rate should match the rate of bone healing, such that the new tissue can sustain cellular integrity and provide mechanical stability [137]. The degradability of hydrogels is determined by various physical parameters, including the composition, degree of crosslinking and the 3D arrangement with other materials, as well as the microenvironment in which they are used [138].

## 8. Manufacturing Strategies for Hydrogel-Based Scaffolds for BTE

Although design strategies have significantly improved hydrogel characteristics, creating a vascularized structure mimicking the native tissue remains a significant challenge. Several microfabrication techniques, such as 3D printing, have been developed to create 3D structures that overcome the challenge of vascularization as well as providing the required porosities that promote cellular ingrowth [115,139]. 3D printing techniques for various hydrogel applications are classified as: (a) laser printing, (b) extrusion printing, and (c) inkjet-based printing [140]. These three fabrication techniques can also efficiently print hydrogels with other blended materials (including cells, growth factors, or materials to enhance the structural integrity) in the desired shape or architecture.

### 8.1. Laser Printing

**Figure 5:**
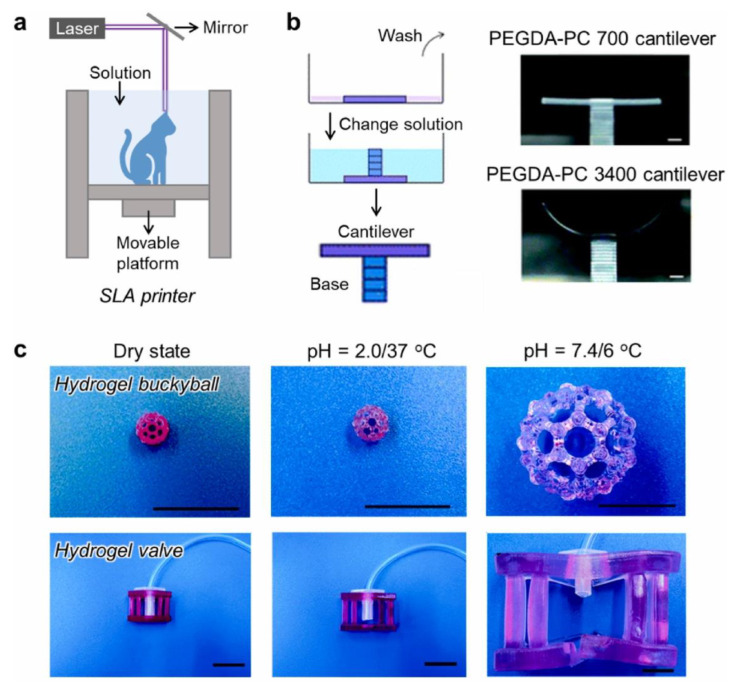
(**a**) Schematic of SLA 3D printer. (**b**) SLA printed hydrogel cantilevers with different molecular weight (700/3400 Da) of PEGDA. Scale bar, 1 mm. (**c**) SLA printed hydrogel buckyball and valve at different condition. Scale bar, 2 cm. Reprinted with permission from Elsevier [152].

Various hydrogels including gelatin, collagen, alginate, PEGDA, etc. have been used to fabricate scaffolds using laser-based 3D printing (Figure 5) [141,142,143]. A classical laser-based 3D printer has three basic components: (i) a pulsed laser beam, (ii) a ribbon to print the scaffold, and (iii) a substrate to collect the printed materials [144]. The laser acts on the absorbing layer of the ribbon to drive the hydrogel by high-pressure gas towards the collector side [139]. A combination of cells and hydrogels are used as bioinks [145] for scaffold fabrication. Laser-based printing technology can print hydrogel-based scaffolds of the desired geometry with micrometer resolution whilst avoiding damage to the cells [139,144,146,147]. Laser bioprinting has been performed in vivo to deposit nano-hydroxyapatite onto rodent calvarial defects with the assistance of computer aided design (CAD) and computer aided manufacture (CAM) workstation. Despite heterogeneous results for bone formation, this work showed the possibility of in vivo laser bioprinting to repair critical size bone damage of a critical size [148]. In vivo laser bioprinting was also attempted by depositing mesenchymal stromal cells onto mice calvarial defects with two differed patterns-a ring or disk-along with nano-hydroxyapatite and collagen. It was discovered that the printing patterns had effects on the cellular arrangement, which in turn influenced bone regeneration [149]. Guene and colleagues (2011) printed mesenchymal stem cells in an alginate hydrogel coated on the donor ribbon [150]. They verified that laser printing caused minimal cell damage and that the printed bone graft exhibited osteogenic and chondrogenic differentiation. Laser bioprinting was also used to print endothelial cell bioinks with a defined pattern onto mesenchymal stem cells seeded collagen hydrogels. The printed endothelial cells were then overlayed by another layer of collagen hydrogel containing vascular endothelial growth factor. This architecture achieved by laser printing showed formation of microvascular network [151].

### 8.2. Extrusion Printing

**Figure 6:**
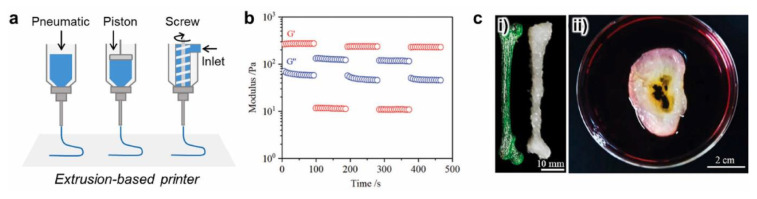
(**a**) Schematic of extrusion-based 3D printers. (**b**) Reversible gel-sol transition of particular gel-based ink under cyclic shearing strains of 1% (G′ > G″) and 10% (G′ < G″). (**c**) i. A rat-size thigh-bone and ii. a human-size ear model fabricated by extrusion-based 3D printing. Reprinted with permission from Elsevier [152].

Extrusion-based 3D printing typically employs three dispensing techniques (pneumatic, piston, and screw dispensers) to distribute the biomaterials onto a substrate (Figure 6) [139]. Extrusion-based 3D bioprinting is compatible with a range of crosslinking methods and is well suited to shear-thinning materials including alginate and PEG-based hybrid hydrogels, and hybrids of gelatin hydrogel such as GelMA [153,154,155]. Alginate and polycaprolactone remain the predominant choices for extrusion bioprinting, followed by GelMA, and most work having been dedicated to bone and cartilage [156]. Extrusion bioprinting has also been found feasible to print hydrogel-based bioinks incorporating bone marrow stromal cells in alginate or Lutrol F127 (copolymer of polyethylene oxide and polypropylene oxide). The printed cells not only survived the extrusion process but also exhibited expression of the osteogenic marker alkaline phosphatase [157]. Although simple 3D structures can be rapidly printed with sufficient resolution, manufacturing complex 3D structures remains challenging.

UV irradiation is widely applied in situ to rapidly cure the printed hydrogel to achieve the desired shape. In one example, the printed ink composed of alginate and acrylamide was exposed to UV irradiation which then initiated photo-polymerisation and crosslinking of acrylamide. The resulting hydrogels were further strengthened by being immersed in CaCl_2_ solution which physically crosslinked the alginate chains [81]. The viscosity of hydrogel inks consisting of alginate and polyethylene glycol diacrylate can be adjusted by the addition of laponite. The ink exhibited shearing thinning behaviour in the extrusion cartridge and recovered its viscosity once it exited the nozzle. Due to the viscosity, no support was required for the printed hydrogel inks to form complex constructs such as a mesh of multiple layers, artificial ears, and artificial noses. The printed hydrogel also demonstrated excellent mechanical toughness [158]. The structural interactions between the two polymer components also proved to be important. As in double network hydrogels, interactions between the two network polymers guarantee that damage in one network can be supported by the other network, leading to toughness enhancement for the overall system [159]. Meanwhile, such an interaction also contributes to the special stress relaxation behaviour of double network hydrogels which can be mathematically described by two exponential models [160]. In another work, chitosan was mixed with alginate and upon extrusion printing the mixture was sprayed with hydrochloride acid which protonated the –NH_2_ groups in the chitosan. The positively charged chitosan was then complexed with negatively charged alginate. The printed hydrogel was shown to support the adhesion and proliferation of the seeded human adipose derived stem cells, making this hydrogel system a candidate for tissue engineering scaffold [161].

### 8.3. Inkjet Printing

**Figure 7:**
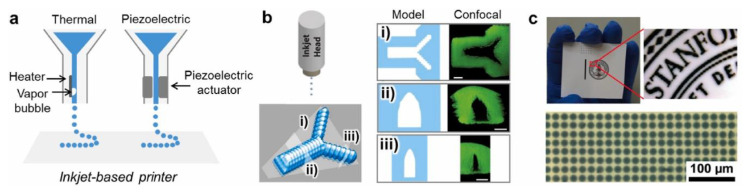
(**a**) Schematic of inkjet-based 3D printer. (**b**) Model and confocal slices at relative positions of a inkjet-based 3D printed microvasculature. Scale bar, 200 µm. (**c**) Micropatterned conducting hydrogel by inkjet-based 3D printing. Reprinted with permission from Elsevier [152].

Inkjet printing is a key technique in the field of customized polymer deposition [162,163]. It is a non-contact reprographic technique that can acquire and reproduce digital data onto a substrate through ink droplets [162]. This technique applies either a drop-on-demand jetting system or a continuous jetting system to 3D print hydrogel-based scaffolds (Figure 7). The inkjet-based printing method offers high fidelity and resolution (50–500 µm) to manufacture complex 3D structures [164]. The droplets formation in inkjet printing can be achieved by either thermal or piezoelectric forces. The thermal printer is reported to not harm the cell survival since it applies heat for only a few microseconds, which leads to a small temperature increase in the bioink above the ambient [165]. Piezoelectric inkjet printers apply a voltage on piezoelectric crystals or ceramics to generate corresponding actuations that can control the ejection of the bioink [166]. With thermal inkjet printers, acrylated polyethylene glycol can be mixed with either acrylated peptide [167] or GelMA [168] to form hydrogel-based scaffolds via in situ photo-polymerisation during the printing process. Human mesenchymal stem cells have been mixed with these hydrogels and printed simultaneously, exhibiting improved osteogenic and chondrogenic differentiation. On the other hand, piezoelectric inkjet printers have been used to perform drop-on-demand printing of human fibroblast cells [169] and Hep G2 hepatocytes onto collagen hydrogels [170]. In the latter work, the effects of surfactant and mild stirring on cell viability and printing reliability were assessed [170]. Inkjet printing can also help to generate controlled hybrid constructs, in one work, rabbit articular chondrocytes were printed with a hydrogel consisting of collagen and fibrin onto electro-spun polycaprolactone. The layer of the cell-laden hydrogel and polycaprolactone fibres were printed alternately, and the hybrid structure demonstrated elevated mechanical properties and cartilage tissue formation [171].

There are several other manufacturing strategies that can be employed to construct 3D hydrogels scaffolds. Photo-lithography is an efficient technique for direct patterning of 3D complex heterogeneous gel structures. This approach utilizes light to crosslink the hydrogel via a photoinitiator [172,173,174]. Photomask-based photolithography technology transmits the light through a mask in a desired pattern and transfers this pattern to the hydrogel [115]. In contrast, maskless photolithography (or stereolithography) is a type of solid freeform fabrication (SFF) that manufactures scaffolds of various materials, including hydrogel, through a stepwise distribution of materials and/or energy [175]. This technique can fabricate 3D scaffolds of distributed geometry with high accuracy and resolution, and with a variety of mechanical properties [139,173,176]. This technique can also create blood vessel-shaped channels to allow for pre-vascularization [177].

### 8.4. Hydrogel-Based In Vitro and In Vivo Bone Tissue Regeneration

Several studies demonstrate in vitro cellular adhesion, migration, proliferation, osteogenic differentiation, and osteogenic gene expression utilizing hydrogels. The combination of biodegradable polymers and bioactive organic and inorganic materials are common strategies to improve the base characteristics of both synthetic and natural hydrogels. These strategies, including the aim of the modification and its effect are summarised in Table 1.

## 9. Hydrogel-Based Bone Tissue Regeneration for Segmental Mandibular Defect Repair

Hydrogels have been widely studied as scaffold materials for cellular support [194]. Although numerous in vitro and in vivo studies have been published, few incorporate load-bearing bone defect models. The broad array of different hydrogels and lack of uniform strategies for their application, many derived from in vitro rather than robust clinical testing, suggest that this approach is still in the early stages of development and that its clinical relevance remains to be proven. Studies have utilized a variety of stem cells, others did not include cells, and only a few assessed neovascularization, which is important for normal bone regeneration. Furthermore, the in vivo studies discussed in Table 1 were conducted in small animal models that are unlikely to resemble the regeneration of segmental mandibular defects in humans.

The anatomical structure of the mandible differs from that of long bones. While bones such as the femur are supported at both ends along the axis, each hemimandible is supported only at one end. There is a significant difference in the loading pattern between the mandible and long bones, with long bones predominantly undergoing compressive loading along their axis, where the mandible undergoes bending loading, with high cantilever forces. Furthermore, the mandible also contains specialized structures such as teeth. Although there is promising data supporting the application of hydrogels in long bones and calvarial bone, these structural differences make hydrogels, in isolation, poorly suited to mandibular defect reconstruction.

This review has highlighted the application of several hydrogels or their composites for regenerating bone tissue, both in vitro and in vivo. As depicted in Table 1, some hydrogels show promise in vitro, and others from animal modelling. In vitro studies are not always translatable to in vivo, as it is not possible to completely mimic the physiological environment. For example, this is not always possible to create an in vivo or living tissue-like environment that would reproduce similar loading patterns on the hydrogels or generate a comparable critical-sized defect. Furthermore, in vitro testing of a given hydrogel is usually done in a controlled environment or system. However, in living animals, similar testing of the given hydrogel is not always controllable, with animal behaviour and physiology having an impact on successful translation of the in vitro results. Notwithstanding these limitations, several in vitro studies presently discussed have indicated hydrogel-based (e.g., alginate, chitosan, collagen, and GelMA) support of cellular proliferation, adhesion, osteogenic differentiation, and degradation.

Hydrogels and modified variants have low mechanical strength compared to cortical bone [195]. The concept of incorporating hydrogels into a stronger biomaterial structure before implantation is appealing and ideally, such a scaffold should have a biomechanical profile similar to bone. Gugala et al. (2002) [194] employed an autogenic cancellous bone graft and a porous (50–70 µm) support structure composed of poly(L/DL-lactide) (80/20%) to heal a 4 cm long segmental tibial defect in a sheep model. The approach of Gugala et al. (2002) [194] was a pre-cursor to the concept of incorporating a scaffold material with a bioactive filler (i.e., hydrogel) supplemented with autogenic bone cells. These hybrid scaffolds can support cell growth, proliferation, and vascularization [196], and a range of polymeric and metallic materials could be used as structural supports, such as poly-ether-ether-ketone (PEEK), poly-ether-ketone-ketone (PEKK), polycaprolactone (PCL), titanium, bioactive ceramics and bioglasses as structural supports. Among these structural biomaterials mentioned above, both PEEK and PEKK have been proven biocompatibility and an appropriate elastic modulus. However, low bio-integration is a significant limitation of these two biomaterials which requires improvement. PEEK’s low bio-integration may be overcome through several modification techniques such as plasma immersion ion implantation [197,198,199,200,201,202], chemical treatment using hydroxylation, carboxylation, amination [201], or surface coating with hydroxyapatite, titanium, gold, or titanium oxide [201]. Polycaprolactone is another proven biomaterial that is reported to act synergistically with hydrogels [185] and growth factors [203] for bone regeneration, however, its mechanical properties are insufficient for the load bearing zones for a given bone, including mandibles.

**Figure 8:**
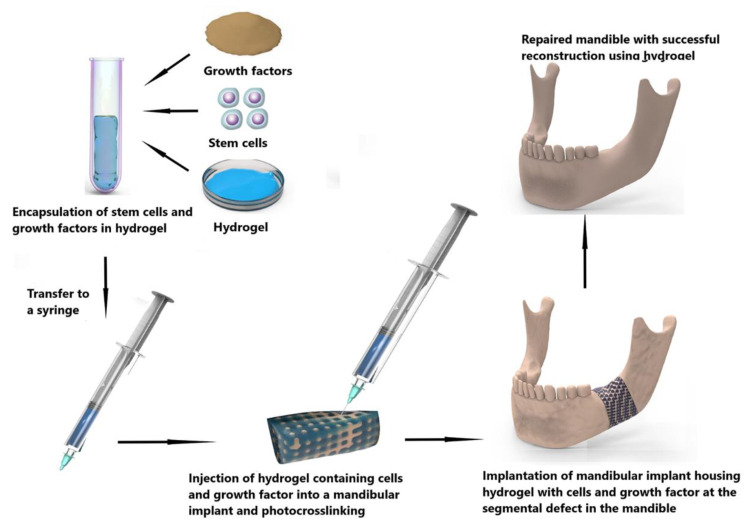
Combination of scaffold made of different biomaterials and hydrogel containing stem cells, and growth factors to repair a segmental mandibular defect.

**Figure 9:**
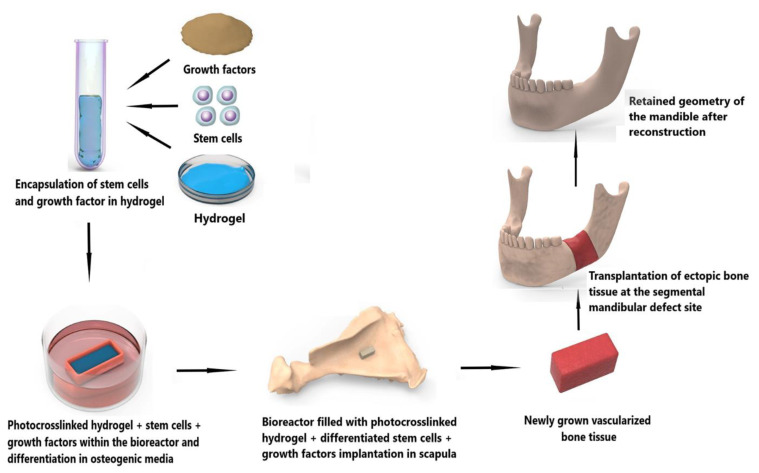
Application of hydrogel, stem cells, and growth factors to create customized ectopic bone with a goal to repair a segmental mandibular defect.

Bioactive constituents could also be incorporated into the hydrogel-scaffold construct before gelation. Numerous studies suggest that hydroxyapatite, tricalcium phosphate (β-TCP), chondroitin sulphate, BMP-2, and BMP-4 promote osteoinduction, osteoconduction, and osteogenesis [190,193,204,205]. The data from Table 1 supports calcium phosphate (hydroxyapatite or β-TCP) and/or BMP-2/4 as being the most evidence-based additions to hydrogels. Whether stem cells are essential, remains to be seen, and although such a construct could be implanted directly to the mandible, in the context of oral cancer, the approach is likely to fail because of the exposure to oral bacteria, poor oral mucosal tissue integrity, and the frequent need for adjuvant radiotherapy. An alternative is to combine osteogenically differentiated stem cells, growth factors, and a suitable hydrogel which can then be injected into a customized porous scaffold made of a non-hydrogel material before being crosslinked. This combined scaffold-cell-growth factor-hydrogel construct can then be applied to a segmental defect for subsequent bone repair (Figure 8). Another alternative is to use an in vivo bioreactor [206] to vascularise the construct and allow a period of osteogenesis within the body. Microvascular surgery can then be used to auto-transplant the living construct to the defect. When exposed to the oral cavity, the vascularised construct will have an innate capacity to self-heal and osseointegrate with the native bone (Figure 9).

Although theoretically possible, the concepts mentioned above for hydrogel-based reconstruction of segmental mandibular defects must be refined through extensive large animal trials such that the biomechanical, osteoinductive, osteoconductive and osseointegrative properties can be optimised.

## 10. Conclusions and Future Perspectives

To date, limited in vivo data exists on the use of hydrogels for bony maxillofacial reconstruction. Notably, no hydrogel has demonstrated efficacy for critical sized bone defects of the mandible. Furthermore, many studies conducted to date have been limited to short-term investigations of cellular support or in acellular environments, so the feasibility for long-term clinical applications remain uncertain. Importantly, most in vivo studies of hydrogels, including chondroitin sulphate combinations and silk nanofiber combinations, have been limited to non-load-bearing bone defect models. Whilst the literature has demonstrated the capacity for such scaffolds to regenerate bone, further research using a load-bearing model is vital.

Notwithstanding the need for further clinically relevant investigation, there exists several barriers to clinical translation of hydrogel-based therapies for bone regeneration. First and foremost, biomaterials without chemical or structural or molecular modification employed for tissue engineering often have limited bioactivity [207]. Therefore, supplementary biologics are often required to be added to the primary scaffold in vitro to enable targeted cellular adhesion, proliferation, gene expression and differentiation. Moreover, the physical interactions of tissue-engineered constructs with the native microenvironment are largely unknown with regard to the regulation of growth, differentiation, and metabolism of progenitor cells [208]. Furthermore, in vivo immunogenic reactions have hindered translation with much research in bone regeneration being directed solely by material scientists and engineers with limited experience of the clinical application for this technology. Designing smart and intelligent nanocomposite scaffolds is also highly essential to improve the potential scaffolds to have the self-healing capabilities necessary for tissue regeneration [209]. Recently, nanofibers of various polymeric biomaterials, for example chitosan hydrogel, become an attractive alternative for a number of biomedical application, including tissue engineering. These nanofibers possess the ability to form networks of fiber mesh with suitable porosity and interconnectivity that enhance their application in engineering complex tissues, including bone [210]. In other areas of tissue engineering, clinician-led approaches have yielded highly translatable outcomes in terms of implantable hollow organs [211]. Although multiple manufacturing strategies to fabricate biomaterial scaffolds have been developed, there remains a lack of consistency and reproducibility in pre-clinical scaffold development for bone tissue engineering and generally insufficient evidence to support their use [207].

Other considerations unique to maxillofacial reconstruction includes the need to satisfy specific biomechanical requirements in relation to the cantilever forces experienced by the mandible during mastication. Guilak et al. (2014) [208] describes how altered biomechanical factors influence bone remodelling of repaired tissue and our incomplete understanding of the material properties for novel scaffolds when implanted in vivo. Of specific concern for the specialty of head and neck surgery is the lack of investigations for into hydrogel-based regeneration for segmental mandibular defects. Further site-specific investigations are needed to examine the potential of hydrogel scaffolds for use in maxillofacial reconstruction.

## Figures and Tables

**Table 1:** Applications of different hydrogels in various in vitro and in vivo bone regeneration studies.

Base Material/s	Modification (Reference)	Control	Aim of Modification	In Vitro or In Vivo (Model Used)	Outcomes Achieved	Comment/Evidence
Alginate-Gelatin	Bioactive glass [178]	Alginate only	Osteogenesis + Increase strength	In vitro (BMSCs)	Osteogenic differentiation with/without bioactive glass in absence of osteogenic stimulants Low osteogenic gene expression Slow degradation Enhanced apatite formation Increased mechanical strength	Osteogenic supplement is essential for upregulating gene expression. Evidence: Low
Oxidized sodium alginate–N-succinyl chitosan	RGD grafting [179]	Hydrogel without cells	Reduce hydrophilicity+ Endothelial differentiation + Osteogenic differentiation	In vitro (BMSCs)	Slow degradation Uniform distribution of pores Chitosan serves as a skeleton for hydrogel networks to increase compressive strength Enhanced cellular response Promote endothelial and osteogenic differentiation	Lack of gene expression data for both endothelial and osteogenic differentiation. Evidence: Low
Glycol-chitosan-Hyaluronic acid	Nano-HA [180]	-	Increase the enzymatic degradation + Osteogenesis	In vitro (MC-3T3-E1 cells)	Porous scaffold Faster enzymatic biodegradability, essential to provide enough space for new bone formation Good cellular attachment and distribution	No information on cellular proliferation, osteogenic differentiation, mechanical strength of the scaffold. Evidence: Low
Methacrylated glycol chitosan (MeGC)	Collagen [181]	MeGC only	Increase strength + Osteogenesis	In vitro (BMSCs, seeded either on surface or encapsulation)	Increased compressive strength Slow degradation Enhanced cellular attachment, distribution, and proliferation Osteogenic differentiation Good mineral deposition	Long-term in vitro study is required to evaluate the degradability and osteogenic gene expression beyond the 21 days. Evidence: Low
Titanium	Surface coating with GelMA and HA [182]	Titanium only	Improved osseointegration	In vitro (without cells)	Enhanced osseointegration claimed	Appropriate model required for evaluating osseointegration Evidence: Low
GelMA	Poly(ethylene glycol) diacrylate (PEGDA), acryloyl-6-aminocaproic acid (A6ACA) and calcium phosphate [183]	Non-mineralized GelMA	Osteogenic differentiation	In vitro (Human induced pluripotent stem cells-hiPSCs)	Osteogenic differentiation of hiPSCs Slow degradation	Evidence: Low
GelMA	Gold nanoparticles [184]	GelMA only and blank control	Enhance osteogenesis	In vitro (adipose-derived stem cells-ADSCs) In vivo (rabbit calvarial defect)	ADSC proliferation Osteogenic differentiation New bone tissue formation	Study in load-bearing bone is required. Evidence: Medium
Alginate-gelatin- Nano-HA	PCL scaffold [185]	Hydrogel-loaded PCL scaffold without cells	Osteogenesis Improving the bioactivity	In vitro (hMSCs)	Mineralisation (apatite)	Evidence: Low
Alginate	Beta-tricalcium phosphate (β-TCP) [186]	None	Osteogenic differentiation	In vitro (MSCs) In vivo (subcutaneous implantation in nude mice)	Improved compressive strength with higher concentration of alginate (2% vs. 1%) Osteogenic differentiation. Osteocalcin gene expression in vitro Calcified tissue deposition on scaffold surface	Cellular behaviour such as cell viability, proliferation in response to the hydrogel system has not been tested Lack of proper control group to compare the findings Reproduction in bone defect model required. Evidence: Medium
Chitosan/β-glycerophosphate	Collagen Type I [187]	Uncoated group for in vitro study and Chitosan/β-glycerophosphate only for In vivo study	Reduces β-glycerophosphate related toxicity Osteogenic differentiation of MSCs	In vitro (Mouse-derived green fluorescent protein-labelled MSCs) In vivo (subcutaneous implantation in nude mice)	Good biocompatibility MSCs maintained their typical shape and phenotypes within the hydrogel Possesses significantly higher cell viability compared to the group without collagen type I Maintain the consistency of increasing level of ALP activity Matrix mineralization and formation of trabeculae	Bone defect model required. Evidence: Medium
Gels based on glyco-nucleo-lipids containing a fluorinated carbon chain (GNF)	Collagen type I [12]	GNF only	Osteogenesis	Both in vitro (hADSCs) and in vivo (subcutaneous implantation of hADSCs encapsulated in the scaffold in nude mice)	Improves the biological properties of the hydrogel as cell culture support Long-term survival of hADSCs in vivo Formation of lamellar osteoid tissue Differentiation into osteoblast	Bone defect model required. Evidence: Medium
Carboxymethyl chitosan (CMCh)	amorphous calcium phosphate (ACP) [188]	Without the CMCh-ACP hybrid gel	Osteogenesis	Both in vitro (BMP-9 induced Human HEK-293 cells) and in vivo (Subcutaneous implantation in mouse model)	Osteoinduction Ectopic bone formation	Evidence: Medium
Peptide-modified alginate	Bone morphogenetic protein-2 (BMP-2) [189]	Nano-fiber mesh without alginate and BMP-2	Osteoinduction	In vivo (Femoral segmental defect in rat)	Improved bone volume and bone density Improved mechanical properties (torque and torsional stiffness) Angiogenesis	Evidence: Medium
Chondroitin sulphate and maleimido terminated polyethylene glycol (PEG-AMI)	BMP-4 [190]	Blank and hydrogel only	Osteoinduction	In vivo (Cranial defect in rat)	Defect repaired by new bone tissue Angiogenesis	Load-bearing bone defect in large animal model required. Evidence: Medium
PEG-PCL-PEG copolymer, and collagen	Nano-HA [191]	Blank control	Osteoinduction	In vivo (rabbit cranial defect)	Osteogenesis	Load-bearing bone model required. Evidence: Medium
Silk nanofiber (SNF)	HA nano particles [192]	Cell only for in vitro and SF for in vivo study	Osteogenesis	Both in vitro (rat BMSCs (rBMSCs) and in vivo (Rat calvarial defect)	Good cellular response to the hydrogel Bone formation with good mineralisation Improved bone volume, bone volume/total volume ratio, trabecular number, and trabecular thickness for new bone formation in scaffold containing HA	HA played a vital role in forming new bone tissue. Load-bearing bone defect required. Evidence: Medium
Silk nanofibers and HA	Deferoxamine (DFO) and BMP-2 [193]	Blank control and SNF/HA only	Neovascularization Bone formation	Both in vitro (rBMSCs) and in vivo (Rat calvarial defect)	Progressive bone growth in the periphery of the defect DFO stimulated regeneration of osteoid Early vascularization induced by both DFO and BMP-2	Require large animal model Evidence: Medium

Evidence Definition: Very Low—no effect; Low—Effect demonstrated by single study in vitro, Medium—multiple studies in vitro or single study in vivo, High—multiple studies in vitro and single study in vivo, Very high—effect demonstrated by multiple studies in vivo.

## Data Availability

No datasets were generated for this review article.
